# Searching for an Accurate Marker-Based Prediction of an Individual Quantitative Trait in Molecular Plant Breeding

**DOI:** 10.3389/fpls.2017.01182

**Published:** 2017-07-06

**Authors:** Yong-Bi Fu, Mo-Hua Yang, Fangqin Zeng, Bill Biligetu

**Affiliations:** ^1^Plant Gene Resources of Canada, Saskatoon Research and Development Centre, Agriculture and Agri-Food Canada, SaskatoonSK, Canada; ^2^College of Forestry, Central South University of Forestry and TechnologyChangsha, China; ^3^Department of Plant Sciences, University of Saskatchewan, SaskatoonSK, Canada

**Keywords:** quantitative trait, RNA-Seq, functional marker, breeding, marker-assisted selection, genomic selection, trait-specific marker selection

## Abstract

Molecular plant breeding with the aid of molecular markers has played an important role in modern plant breeding over the last two decades. Many marker-based predictions for quantitative traits have been made to enhance parental selection, but the trait prediction accuracy remains generally low, even with the aid of dense, genome-wide SNP markers. To search for more accurate trait-specific prediction with informative SNP markers, we conducted a literature review on the prediction issues in molecular plant breeding and on the applicability of an RNA-Seq technique for developing function-associated specific trait (FAST) SNP markers. To understand whether and how FAST SNP markers could enhance trait prediction, we also performed a theoretical reasoning on the effectiveness of these markers in a trait-specific prediction, and verified the reasoning through computer simulation. To the end, the search yielded an alternative to regular genomic selection with FAST SNP markers that could be explored to achieve more accurate trait-specific prediction. Continuous search for better alternatives is encouraged to enhance marker-based predictions for an individual quantitative trait in molecular plant breeding.

## Introduction

Molecular plant breeding with the aid of molecular markers has played an important role in modern plant breeding over the last two decades (Moose and Mumm, 2008). Many useful markers have been developed and applied to enhance parental selection in breeding programs (e.g., Randhawa et al., 2013; Grover and Sharma, 2016). Recent advances in next-generation sequencing (NGS) technology (Varshney et al., 2009; Metzker, 2010) have helped to generate abundant low-cost molecular markers and make the molecular markers more useful and informative for plant breeding (Varshney et al., 2014). Currently, there are two major approaches applied for molecular breeding: marker-assisted selection (MAS) and genomic selection (GS or Genome-wide selection) (Jiang, 2013). Traditional MAS is based on the selection of statistically significant, marker-trait associations and enhances parental selection for recessive trait and disease resistance in conventional breeding program without observing phenotypic variation in the traits. However, traditional MAS is not well-suited for complex traits controlled by many genes (Beavis, 1998). GS, introduced first in animal breeding (Meuwissen et al., 2001), estimates genome-wide marker effects and uses the estimates to predict individual genetic potential (i.e., genomic estimated breeding values). Studies have shown that GS outperforms MAS in parental selection, particularly for those complex traits controlled by a large number of genes (e.g., see Bernardo and Yu, 2007; Massman et al., 2013; Sorrells, 2015; Liu et al., 2016). However, GS applications are not lacking of technical issues and usually display low accuracies of predicting trait performances (Jannink et al., 2010; Windhausen et al., 2012; Riedelsheimer et al., 2013; Bassi et al., 2016; Rabier et al., 2016). Thus, improving trait prediction accuracy is one of the active research areas in molecular plant breeding, and the development of genome-wide informative markers through NGS remains a major theme of research (Yang et al., 2015).

RNA-Sequencing (or RNA-Seq) is a recently developed genomic approach for transcriptome profiling, can be applied to study each transcript of genes affecting a trait at a developmental stage, and has opened many avenues to develop informative markers associated with genes controlling genetically complex traits of agronomical importance (Wang et al., 2009; Ozsolak and Milos, 2011). Here we attempt to search for alternatives to GS for more accurate trait prediction through a literature review on the prediction issues in molecular plant breeding and on the applicability of an RNA-Seq technique for developing function-associated specific trait (FAST) SNP markers. We also perform a theoretical reasoning on whether and how FAST SNP markers could enhance individual trait prediction and verify the reasoning through computer simulation. It is our hope that this effort would seed an alternative with specific trait SNP markers that can be explored to achieve more accurate prediction for a quantitative trait in molecular plant breeding.

## Molecular Plant Breeding and its Limitations

Molecular plant breeding is generally termed as the application of molecular markers to improve the characters of interest in plants (Xu, 2010; Jiang, 2013), and is one of the modern breeding strategies with the potential to accelerate breeding efficiency (Moose and Mumm, 2008). Conventional plant breeding is largely relied on phenotypic selection through cycles of crossing and selection and requires substantial breeding efforts with more than 10 years to develop an improved variety. The major challenge lies in the low efficiency of phenotypic selection for desirable traits of quantitative nature such as yield and disease resistance that are controlled by many genes of small effects and their interactions with environments. Thus, efficient methods have been searched to improve the selection of individual plants with desired traits, including MAS.

The idea for the use of markers to assist plant selection could date back to the association analysis done by Sax (1923) between seed color (monogenic trait) and seed weight (polygenic, quantitatively inherited trait) in beans (*Phaseolus vulgaris* L.) and the promotion made by Thoday (1961) on the mapping of polygenic traits with the help of monogenic morphological markers. Although allozyme markers were applied in the early 1980s to identify genotypes, the idea of MAS was not flourished until the development of the first DNA-based genetic markers, restriction fragment length polymorphisms (Botstein et al., 1980). Since then, large efforts have been made to develop molecular markers such as random-amplified polymorphic DNAs, amplified fragment length polymorphisms, simple sequence repeats or single nucleotide polymorphisms (Grover and Sharma, 2016). Such advance in molecular markers not only stimulated the theoretical investigation on MAS efficiency (e.g., see Lande and Thompson, 1990), but also made the MAS practically feasible to complement and enhance the conventional plant breeding (Moose and Mumm, 2008). Accordingly, many MAS techniques have been developed, including marker-assisted backcrossing (MABC), marker-assisted recurrent selection (MARS), and GS (Jiang, 2013). With the recent advance in NGS and the development of genome-wide SNP markers, GS will be more efficient, even in MABC and MARS. These technical developments have made the molecular breeding a standard practice complementary to conventional breeding to improve traits with complex genetic bases (Moose and Mumm, 2008).

As expected with the promise of MAS, several reviews have confirmed that the research and use of molecular markers in plant breeding have continued to increase in the public and private sectors, particularly since the 2000s (Beavis, 1998; Holland, 2004; Collard and Mackill, 2008; Xu and Crouch, 2008; Brumlop and Finckh, 2011; Boopathi, 2013). Successful stories for MAS applications are not lacking (e.g., see Collard and Mackill, 2008; Boopathi, 2013; Randhawa et al., 2013). For example, many molecular markers were deployed to assist selection for disease resistance, agronomic and quality traits in several wheat (*Triticum* spp.) cultivars released for commercial cultivation in Canada (Randhawa et al., 2013). However, MAS applications mainly focused on simply inherited traits, such as monogenic or oligogenic resistance to diseases/pests, although quantitative traits were also involved (Collard and Mackill, 2008). Also, these MAS applications have not achieved the results as expected previously in terms of extent and success (e.g., release of commercial cultivars). For example, Collard and Mackill (2008) listed 10 reasons for the low impact of MAS in general and Jiang (2013) highlighted seven issues associated with MAS applications. Among them are (1) not all markers are breeder-friendly, (2) not all markers can be applicable across populations due to lack of marker polymorphism or reliable marker-trait association, (3) false selection may occur due to recombination between the markers and the genes or quantitative trait loci (QTL) of interest, and (4) imprecise estimates of QTL locations and effects result in slower progress than expected. Improvement of most agronomic traits that are of complicated inheritance and economic importance like yield and quality is still a great challenge for MAS including the newly developed GS (Jannink et al., 2010). Jiang (2013) indicated that MAS is not universally or necessarily advantageous, at least from the viewpoint of a plant breeder.

**FIGURE 1:**
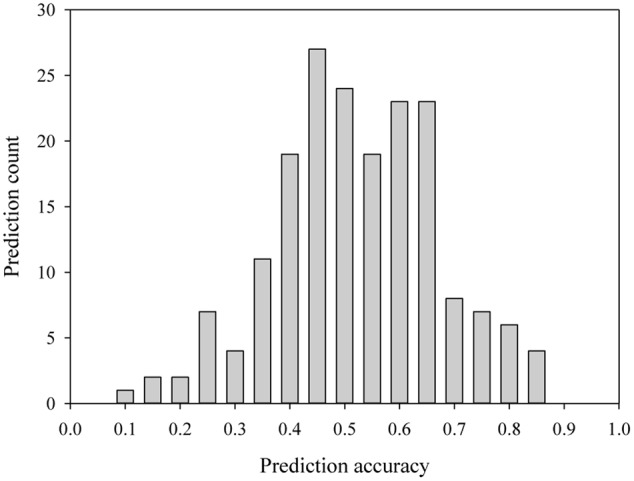
The distribution of 187 prediction accuracies from genomic selection for several traits in crop and tree species, as reported in a selection of 31 peer-review journal publications from 2015 to 2016 (see the Supplementary Table S1).

Last several years have seen increased researches directed toward GS applications (e.g., see Spindel et al., 2015; Bassi et al., 2016). With the advances in the development of cost-effective genome wide markers, it is no doubt that some of the old challenges faced with the MAS applications can be addressed (Jannink et al., 2010). Several applications have demonstrated its usefulness in actual plant breeding programs (e.g., see Sorrells, 2015; Bassi et al., 2016). However, some marked features of GS in plant breeding have also started to emerge (Windhausen et al., 2012; Riedelsheimer et al., 2013; Spindel et al., 2015). First, the accuracy of the genome-wide marker prediction on trait performance has a range of estimates, but is generally low, depending on many factors including crop, trait, marker, training population, GS model, and environment (Rabier et al., 2016). To update the current status of prediction accuracy, we selected 31 peer-review journal publications from 2015 to July of 2016 that reported genomic selections in crop and tree species, and obtained 187 genomic predictions of trait performance with a range of 0.05 to 0.83 and a mean of 0.50 (**Figure 1** and Supplementary Table S1). For example, a range of prediction accuracies from 0.31 to 0.63 for several traits were found in rice (*Oryza sativa* L.) (Spindel et al., 2015); 0.14 to 0.58 for spring barley (*Hordeum vulgare* L.) and 0.40 to 0.80 for winter barley in malting quality traits (Schmidt et al., 2016); 0.10 to 0.51 in maize (*Zea mays* L.) root traits (Pace et al., 2015); and 0.39 to 0.61 in Canola (Jan et al., 2016). Second, some studies have shown that more genomic markers evenly distributed across the genome do not always help to increase the prediction accuracy and as low as 1000 genomic markers can achieve the same level of prediction accuracy for some traits (Spindel et al., 2015; Jan et al., 2016). These features help to explain partly some less optimistic views of GS potential (Bernardo, 2016), and suggest that more research are required on the choice and development of informative genomic markers for GS.

Our analysis of the GS applications with respect to prediction accuracy concurs well with the renewed argument that more research efforts are needed to develop functional markers for MAS, taking advantage of the recent advances in NGS application (Lau et al., 2015; Yang et al., 2015). This realization is not surprising, as the idea for developing functional DNA markers for plant breeding is not new (e.g., see Andersen and Lübberstedt, 2003; Varshney et al., 2005). However, large efforts have been made with limited success, even in major crop species (Iyer-Pascuzzi and McCouch, 2007; Liu Y. et al., 2012; Lau et al., 2015; Yang et al., 2015). The searching for functional markers via QTL and expression QTL (eQTL) analyses (Druka et al., 2010) or gene cloning with limited genomic resources is technically challenging, labor extensive and time consuming (Yang et al., 2015). Acquiring a relevant set of functional or useful markers through genome-wide association mapping (GWAS) is technically possible for marker-based prediction of trait performance, but practically depends highly on the genotyping accuracy and linkage disequilibrium (LD), and is largely limited to the assayed populations in given environments (e.g., see Eichler et al., 2010; Desta and Ortiz, 2014; Spindel et al., 2016). Dr. Hong-Bin Zhang at Texas A&M University has promoted the idea of gene-based breeding system since 2014 and demonstrated substantial gains in trait prediction from gene-based markers in both cotton (*Gossypium hirsutum*; Liu et al., 2017) and maize (Zhang et al., 2017). For example, using 474 Gossypium fiber length genes, they were able to predict fiber length with correlation coefficients ranging from 0.67 to 0.85 (Liu et al., 2017). However, inadequate attention has been paid to gene-based breeding system. This dilemma seems to suggest that a paradigm shift is needed to develop functional or function-associated markers, particularly focusing on specific quantitative traits.

## RNA-Seq and SNP Markers for Specific Traits

RNA-Seq is a recently developed genomic technology using NGS to study transcriptome (Marioni et al., 2008; Nagalakshmi et al., 2008; Wilhelm et al., 2008). The transcriptome is usually defined as the set of all RNA molecules transcribed in an organ or tissue at a particular point of time under a given set of environmental conditions. Generally, RNA-Seq has two major components. First, RNA is purified from a sample of interest and converted to a library of cDNA fragments with adaptors attached to one or both ends. Each cDNA fragment, with or without enriched with PCR amplification, is then sequenced using one of high throughput sequencing methods to obtain short sequences from one end (single-end sequencing) or both ends (pair-end sequencing). Second, a suite of bioinformatics tools are used to process the raw sequence reads, typically 30–400 bp, map the processed sequences to a reference genome or reference transcripts, or *de novo* assemble without the genomic sequence, and analyze the alternative gene spliced transcripts, post-transcriptional modifications, gene fusion, mutations/SNPs and changes in gene expression (Garber et al., 2011; Lopez-Maestre et al., 2016).

RNA-Seq has a number of advantages over hybridization-based microarrays. First, it does not require existing genomic sequence information to identify transcripts, making its application to non-model plants more feasible. Second, the detection of differentially expressed genes is more accurate, sensitive and reproducible, with fewer systematic discrepancies among technical replicates (Marioni et al., 2008; Nagalakshmi et al., 2008). Third, RNA-Seq allows for quantification of the abundance or changes of each transcript in a developmental stage or under a specific treatment condition (Mortazavi et al., 2008), making the study of a complex transcriptome possible. The paired-end tag sequencing strategy of RNA-Seq further improves cDNA sequencing efficiency with expanded length of short reads for better understanding of the dynamic transcriptomes (Fullwood et al., 2009).

A variety of RNA-Seq applications have been found, ranging from transcriptome profiling to gene discovery, alternative splicing analysis and molecular marker development (Conesa et al., 2016). RNA-Seq has been successfully applied to study the transcriptomes of different tissues such as root (Postnikova et al., 2013), leaf (Li et al., 2010), flower (Mantegazza et al., 2014), fruit (Kang et al., 2013; Martínez-López et al., 2014), and seed (Jones and Vodkin, 2013). Also, it has been employed to analyze gene expressions for biotic and abiotic responses like diseases resistance (Kong et al., 2015), drought stress (Kakumanu et al., 2012; Bhardwaj et al., 2015), cold stress (Sinha et al., 2015; Hu et al., 2016) and chemical stress (He et al., 2015). Moreover, RNA-Seq applications have been reported in many plant species such as *Arabidopsis*, rice, maize, as well as non-model species such as soybean [*Glycine max* (L). *Merr.*] and wheat (Jiao et al., 2009; Filichkin et al., 2010; Li et al., 2010; Severin et al., 2010; Ramirez-Gonzalez et al., 2015). These applications have demonstrated its tremendous power in characterizing transcriptomes, as it can detect low-expressed transcripts, splice variants, and novel transcripts (Socquet-Juglard et al., 2013). Therefore, RNA-Seq is now regarded as the latest and most powerful tool for sequencing and profiling of transcriptome (Han et al., 2015; Conesa et al., 2016).

Last several years have seen increased efforts toward the development of SSR and/or SNP markers through RNA-Seq in many organisms (e.g., see Wei et al., 2011; Yang et al., 2011; Salem et al., 2012; Ulloa et al., 2015; Zhou et al., 2016). Abundant RNA-Seq SNP markers have been developed to sample polymorphisms within the transcribed region of all genes associated with many traits. Many of these markers may be function-associated with some traits of interest, differing from those selectively neutral markers, but not necessarily are qualified as functional markers for a specific trait. This may reflect the fact that many RNA-Seq analyses were performed with the goals to generate dense, genome-wide function-associated markers for linkage mapping and association mapping, not necessarily for direct GS application. Also, it is challenging to develop truly functional markers for specific complex traits, as it requires specific RNA-Seq designs for specific traits and the resulting SNP markers are required to verify their associations with causal genes influencing the traits.

However, it is practically feasible to develop FAST SNP markers through specific RNA-Seq designs and the developed markers have a high probability of being functionally relevant when compared to randomly selected polymorphisms. Note that FAST markers are not technically new, but termed here to distinguish them from others. For example, Salem et al. (2012) conducted an RNA-Seq whole-transcriptome analysis of pooled cDNA samples from a population of rainbow trout (*Oncorhynchus mykiss*) selected for improved growth versus unselected genetic cohorts and developed many FAST SNP markers for growth traits for fish breeding. Similarly, Ulloa et al. (2015) performed an RNA-Seq analysis of eight low-growth and eight high-growth Zebrafish (*Danio rerio*) and developed 164 SNPs, five of which were associated with genes affecting fish growth. Chopra et al. (2015) applied an RNA-Seq to develop and validate a set of gene-based SNPs in sorghum (*Sorghum bicolor*) genotypes with contrasting responses to cold stress. Ramirez-Gonzalez et al. (2015) implemented an RNA-Seq analysis of bulked pools sampled from a F2 population to identify 175 putative SNP markers associated with *Yr15*, the yellow rust (*Puccinia striiformis f.sp. tritici*) resistance in wheat germplasm. Clearly, the most successful applications in plants were those using RNA-Seq in combination with bulked segregant analysis (Michelmore et al., 1991; Liu S. et al., 2012). Similarly, this approach has also facilitated the development of the high resolution SNP maps for wheat grain protein content (Trick et al., 2012) and for the fertility restorer genes of cytoplasmic male-sterility in radish (*Raphanus sativus* L.) and onion (*Allium cepa* L.) (Lee et al., 2014; Kim et al., 2015). These successful applications are encouraging for developing FAST SNP markers for individual traits of breeding target.

## Theoretical Reasoning and Computer Simulation

Our literature review indicates the importance of using functional markers to increase the trait prediction accuracy for GS. This should not be surprised, as functional markers should be more informative to acquire genetic effects of causal genes for trait prediction than genome-wide neutral markers (Mackay, 2001). Using more random, non-causal SNP markers can inflate individual genomic relationships and decrease trait prediction accuracy (Spindel et al., 2015; Edwards et al., 2016). Also, functional markers can avoid marker validation like those random markers in different breeding populations and could be gene or trait specific (Lau et al., 2015; Yang et al., 2015). Our review also indicates various challenges in the development of ideal functional markers for specific traits, but shows the feasibility of developing FAST SNP markers through RNA-Seq. Thus, we reasoned that FAST SNP markers may not supersede the ideal functional markers, but should be more informative, to predict genetic effects associated with a given trait than those dense, genome-wide neutral markers. This reasoning is based on two expectations that the extent of LD between FAST SNP markers and causal genes is generally larger than those between genome-wide neutral markers and casual genes, and that the trait prediction accuracy is positively related to LD (Meuwissen et al., 2001; Fernando et al., 2007).

To understand these two expectations, Fernando and his colleagues conducted extensive computer simulations (e.g., Fernando et al., 2007; Kizilkaya et al., 2010) to illustrate the impacts of LD between SNP markers and casual genes on trait predictions of young cattle (*Bos taurus*). In one simulation on an ideal pattern of LD with marker loci either in complete LD or linkage equilibrium with QTL, they found only the prediction method of Bayes-B (Meuwissen et al., 2001) could achieve up to 0.98 prediction accuracy, while the other two methods RR-BLUP and TP-BLUP displayed lower, unstable trait predictions (Fernando et al., 2007). Considering scenarios with more realistic LD patterns for 30 chromosomes with up to 2000 markers each, they found the accuracy of trait prediction by Bayes-B did not increase after 500 markers per chromosome (Fernando et al., 2007). These findings clearly indicate the importance of LD patterns in a trait prediction. To verify their simulated findings, they used actual 50K SNP data of 1,086 purebred (PB) and 924 multibreed (MB) Angus cattle from eight sire breeds, simulated a trait with the heritability of 0.5 controlled by 50, 100, 250, or 500 additive QTL selected randomly from 50K SNPs, and examined five marker panels (mp) with variable levels of LD for genetic evaluation (Kizilkaya et al., 2010). Specifically, for each QTL scenario, mp1 is an ideal case with only QTL genotypes; mp2 is another extreme with both QTL genotypes and equal number of marker loci with the highest linkage disequilibrium (HLD) for each QTL; mp3 reflects the common practice with all genome-wide SNPs, including QTL; mp4 represents a case of markers each having HLD with an QTL; and mp5 reflects a case of markers with all the SNPs minus QTL. The simulated correlations between true and predicted genotypic values by Bayes-B in the PB validation data set are shown in **Table 1**. As expected, the ideal functional markers with QTL genotypes (mp1) displayed the highest prediction accuracies, ranging from 0.72 to 0.95 and increasing with fewer QTL. When there were more than 100 QTL, the highly linked markers (mp4) showed higher prediction accuracies than all genome-wide SNPs including QTL (mp3). Thus, these simulation results are consistent with the two expectations mentioned above for FAST SNPs, as FAST SNPs should approach the behavior of those highly linked markers to QTL (mp4) for a trait predication.

**Table 1:** Comparative simulation results on the accuracies of predicting a quantitative trait with heritability 0.5 by genomic prediction model Bayes-B based on 50K Angus cattle and 36,543 soybean SNP data with respect to QTL scenario and marker panel.

Angus cattle^∗^		Soybean^∗^	
QTL scenario/marker panel^Φ^	Correlation^#^	QTL scenario/marker panel^Φ^	Correlation^#^
*QTL50*		*QTL50*	
mp1: 50 QTL	0.953	mp1: 50 QTL	0.94 (0.01)
mp2: 50 QTL + 50 HLD	0.931	mp2: 50 QTL + 50 HLD	0.93 (0.01)
mp3: 50K SNPs with QTL	0.766	mp3: 36543 SNPs with QTL	0.64 (0.07)
mp4: 50 HLD	0.570	mp4: 50 HLD	0.83 (0.03)
mp5: 50K SNPs - 50 QTL	0.388	mp5: 36543 SNPs - 50 QTL	0.63 (0.07)
		mp6: 100 HLDr2	0.73 (0.06)
		mp7: 100 HLDr2 + 50 rSNP	0.72 (0.06)
*QTL100*		*QTL100*	
mp1: 100 QTL	0.938	mp1: 100 QTL	0.88 (0.02)
mp2: 100 QTL + 100 HLD	0.914	mp2: 100 QTL + 100 HLD	0.87 (0.02)
mp3: 50K SNPs with QTL	0.585	mp3: 36543 SNPs with QTL	0.61 (0.09)
mp4: 100 HLD	0.513	mp4: 100 HLD	0.77 (0.05)
mp5: 50K SNPs - 100 QTL	0.289	mp5: 36543 SNPs - 100 QTL	0.60 (0.09)
		mp6: 200 HLDr2	0.67 (0.07)
		mp7: 200 HLDr2 + 100 rSNP	0.66 (0.08)
*QTL250*		*QTL250*	
mp1: 250 QTL	0.840	mp1: 250 QTL	0.78 (0.03)
mp2: 250 QTL + 250 HLD	0.788	mp2: 250 QTL + 250 HLD	0.77 (0.04)
mp3: 50K SNPs with QTL	0.399	mp3: 36543 SNPs with QTL	0.61 (0.07)
mp4: 250 HLD	0.510	mp4: 250 HLD	0.71 (0.05)
mp5: 50K SNPs - 250 QTL	0.247	mp5: 36543 SNPs - 250 QTL	0.61 (0.07)
		mp6: 500 HLDr2	0.63 (0.06)
		mp7: 500 HLDr2 + 250 rSNP	0.62 (0.06)
*QTL500*		*QTL500*	
mp1: 500 QTL	0.720	mp1: 500 QTL	0.70 (0.06)
mp2: 500 QTL + 500 HLD	0.642	mp2: 500 QTL + 500 HLD	0.70 (0.07)
mp3: 50K SNPs with QTL	0.254	mp3: 36543 SNPs with QTL	0.60 (0.08)
mp4: 500 HLD	0.372	mp4: 500 HLD	0.65 (0.08)
mp5: 50K SNPs - 500 QTL	0.200	mp5: 36543 SNPs - 500 QTL	0.60 (0.08)
		mp6: 1000 HLDr2	0.62 (0.08)
		mp7: 1000 HLDr2 + 500 rSNP	0.61 (0.08)

*^∗^The results for Angus cattle were acquired from Table 2 of Kizilkaya et al. (2010) and those for soybean were obtained from this simulation.*

*^Φ^Both cattle and soybean simulations applied the same QTL scenarios and the first five marker panels (mp), but soybean simulation had two extra panels. For each QTL scenario, mp1 is an ideal case with only QTL genotypes; mp2 is another extreme with both QTL genotypes and equal number of marker loci with the highest linkage disequilibrium (HLD) for each QTL; mp3 reflects the common practice with all SNPs, including QTL; mp4 represents a case of markers each having HLD with an QTL; and mp5 reflects a case of markers with all the SNPs minus QTL; mp6 consists of the marker loci in which two markers are randomly selected from loci with the highest 20 LD values for each QTL (HLDr2), and mp7 considers both mp6 and a set of random SNP markers (rSNP) each falsely representing an QTL.*

*^#^The correlations between true and predicted genotypic values in the validation data sets and their standard deviations in parentheses. Training and validation sets consisted of 924 multibreed and 1086 purebred cattle, respectively, and for soybean, training and validation populations had 400 plants each.*

To confirm the simulation results in Angus cattle, particularly with respect to mp4, we also conducted a computer simulation based on existing SoySNP50K data (Song et al., 2015), following exactly the same simulation approach used by Kizilkaya et al. (2010) in Angus cattle. First, we randomly selected 800 soybean plants from the 18,480 domesticated soybean accessions with 42,509 polymorphic SNP markers. After excluding the scaffold SNPs and minor alleles (of frequency less than 0.05) and replacing missing data with common haplotypes, we obtained a final soybean data for this simulation with 800 plants with 36,543 SNP markers and divided them into half, each representing a training or validation set. Second, we simulated the same four QTL scenarios as in cattle with 50, 100, 250, and 500 additive QTL that were randomly selected from 36,543 SNPs, and applied the same five marker panels (mp1 to mp5) as described above and two additional marker panels (mp6 and mp7). Specifically, mp6 consisted of the marker loci in which two markers were randomly selected from loci with the highest 20 LD values for each QTL (HLDr2), and mp7 included both mp6 and a set of random SNP markers (rSNP) each falsely representing an QTL. Third, we also considered two heritabilities 0.5 and 0.2, and applied two extra genomic selection models (Bayes-C, and RR-BLUP), besides Bayes-B. Fourth, for each marker panel with different QTL scenarios, random select 400 soybean marker data representing as the training population and another 400 plants as the validation population for prediction for five times, we generated five more replicates than Kizilkaya et al. (2010) did to get average correlations between true and predicted trait values in each random selected validation set. The simulation was conducted with a custom R script (R Core Team, 2015) that was specifically developed for this confirmation and is available upon request to the first author. Marker effect estimation and genetic value prediction were made using the BGLR statistical package in R (Pérez-Rodriguez and de los Campos, 2014) implemented with three genomic prediction models [RR-BLUP ( = GBLUP), Bayes-B and Bayes-C] and confirmed with the *rrBLUP mixed.solve* function (Endelman, 2011).

Our simulation not only confirmed those observed in the Angus cattle, but also revealed some interesting patterns of trait prediction (**Table 1** and Supplementary Table S2). First, the patterns of prediction accuracy by Bayes-B in soybean are the same as in cattle for the QTL scenarios of QTL250 and QTL500. In any QTL scenario, soybean functional markers (mp1) always showed the highest accuracies of trait prediction, followed by highly linked markers (mp4) and all genome-wide SNP markers (mp3) (**Table 1**). Also, the patterns of decreased prediction accuracies by functional markers (mp1) with more QTL were also observed in soybean data (**Table 1**). Second, soybean markers with a little relaxed LD to QTL like mp6 or mp7 still displayed higher prediction accuracies than those genome-wide SNP markers (mp3) in any QTL scenarios assayed. For example, for a trait of heritability 0.5 with 100 QTL, 200 HLDr2 markers (mp6) displayed a correlation of 0.67 while all 36,543 SNP markers (mp3) had only a correlation of 0.61 (**Table 1**). Third, several extra patterns of prediction accuracy were also observed in soybean (**Table 1** and Supplementary Table S2). Three different prediction methods did not show much difference in prediction accuracy. The prediction accuracies became lower for a trait of lower heritability. The prediction accuracies using 36,543 SNPs for a trait of heritability 0.2 ranged from 0.48 to 0.53, while those using highly linked markers (mp6 or mp7) ranged from 0.49 to 0.63. All together, these simulation results demonstrated the potential gain in prediction accuracy from the application of FAST SNP markers in molecular breeding. More evenly distributed markers unlinked to causal genes do not enhance, but rather reduce, trait prediction accuracy.

Our simulation on soybean data had a simple goal to reason the potential of FAST SNP markers and thus was not comprehensive. Further detailed simulations are possible to consider all existing marker prediction models, the related parameters associated with QTL genetic model, marker distribution and informativeness, training set and test environment (Heffner et al., 2009, 2010; Zhong et al., 2009; Hickey et al., 2014). However, our simulation results are consistent with several empirical reports from GS analyses that prediction accuracies were higher using only the QTL-linked markers or a subset of informative markers (e.g., Spindel et al., 2015; Thavamanikumar et al., 2015; Arruda et al., 2016; Edwards et al., 2016; Huang et al., 2016; Liu et al., 2016). Thus, the simulations in Angus cattle and soybean, along those empirical reports, provided support for our theoretical reasoning to search for more informative FAST SNP markers through RNA-Seq to improve trait prediction accuracy.

## An Alternative for Individual Trait Prediction

Based on the literature review and theoretical reasoning, we synthesized that FAST SNP markers can be developed through RNA-seq for an individual quantitative trait and applied to increase the trait prediction accuracy. To better utilize this synthesis, we conceived a marker-based and trait-specific strategy as an alternative to regular GS with FAST SNP markers for plant breeders to facilitate parental selection. We termed it as trait-specific marker selection (TSMS) for ease of interpretation and comparison to marker-specific selection and GS. It is our hope that this alternative or its modifications later can provide a useful breeding tool to improve the accuracy of marker-based prediction on individual trait performance.

**FIGURE 2:**
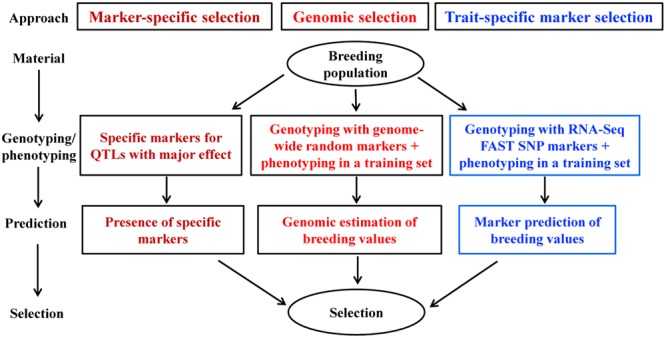
Capturing genes of major and minor effects for a quantitative trait of interest through RNA-Seq function-associated specific trait (FAST) SNP markers in molecular plant breeding. Marker-specific selection uses the specific markers developed from the major QTL of the trait to select desirable parents. Genomic selection applies genome-wide random markers to estimate breeding values in various traits for parental selection. The derived alternative named trait-specific marker selection (TSMS) employs FAST SNP markers developed from RNA-Seq to predict individual performances in a trait of interest for parental selection.

**FIGURE 3:**
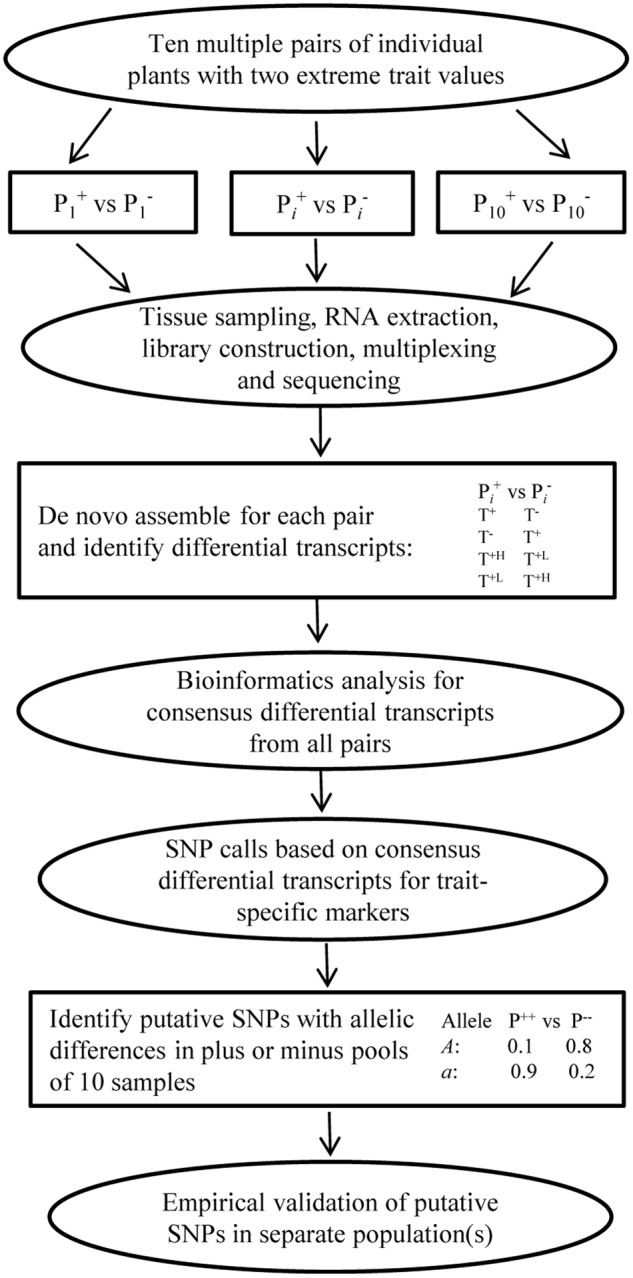
A general procedure for developing plant FAST SNP markers through RNA-Seq. It involves multiple pairs of individual plants with extreme trait values, tissue sampling, RNA-Seq, bioinformatics analysis for differential transcripts for each pair and consensus different transcripts from all the pairs, SNP calls for specific trait markers, putative SNP identification and empirical validation. Identification of a differential transcript in a pair is made based on the presence (T^+^) or absence (T^-^) of a transcript or the difference in abundance (T^+H^ or T^+L^) of the transcript detected in both plants. Putative SNPs are identified with a SNP filter based on the differences in allelic frequency between two trait-extreme sample sets.

**FIGURE 4:**
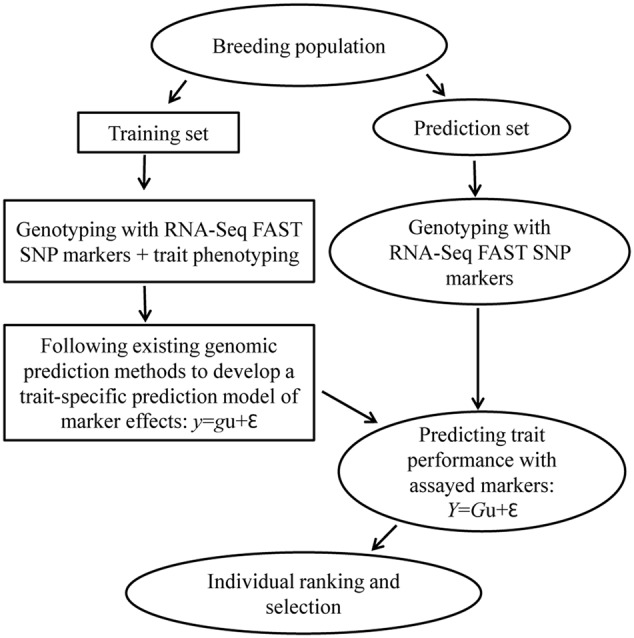
A general procedure for trait performance prediction using RNA-Seq function-associated specific trait (FAST) SNP markers in a breeding population. It follows the same idea of genomic selection to have a training set of breeding materials for both genotyping and phenotyping to develop a trait-specific prediction model of marker effects. The developed prediction model will be applied to predict the trait performance with assayed RNA-Seq FAST SNP markers in the prediction set of breeding population for individual ranking and parental selection. Note that in the prediction equations, *y* or *Y* is the vector of trait values, u is the vector of marker effects, *g* or *G* is the genotype matrix and 𝜀 is the random error vector.

Trait-specific marker selection represents an added option to GS that can be applied to assist parental selection by predicting specific trait breeding values of individual plants in a breeding population through the separate development and application of RNA-Seq FAST SNP markers for specific traits of interest (**Figure 2**). It requires the development and validation of FAST SNP markers for a given trait in other populations, before the application to genotype a breeding population of interest; estimates the marker “effects” in a training set of the breeding population; and applies the estimated marker “effects” to predict trait performance in the same breeding population. Such an approach differs from traditional MAS with QTL-specific markers in genotyping and prediction, but follows the same idea of GS to predict trait performance with RNA-Seq FAST SNP markers, rather than the genome-wide selectively neutral SNP markers. To make the strategy more understandable, we outline the two major components of TSMS in **Figures 3, 4** for developing FAST SNP markers through RNA-Seq technology and for performing SNP marker prediction of breeding values, respectively. The proposed RNA-Seq method (**Figure 3**) considers multiple pairs of individual plants with two extreme trait values and collects their sample tissues at given developmental stages for gene expressions associated with the trait of interest. The collected samples will be subjected to RNA-Seq analysis through RNA extraction, cDNA library preparation, multiplexing with barcoding and cDNA sequencing. The collected RNA-Seq data will be analyzed through *de novo* assembly using various bioinformatics tools to identify differential transcripts for each pair and to generate consensus differential transcripts from all the assayed pairs. Identification of a differential transcript in a pair is made based on the presence or absence of a transcript or the difference in abundance of the transcript detected in both plants. Multiple pairs are used to enhance the reliability of identifying differential transcripts for the trait. Based on the consensus differential transcripts, SNP call will be made from all the samples and the detected SNPs will be filtered to generate putative SNPs for the trait based on the differences in allelic frequency between two trait-extreme sets of assayed samples. An empirical validation of putative SNPs in separate population(s) is required to confirm if the acquired SNP markers are truly associated with the trait performance. The validated SNP markers can be applied to genotype all the breeding materials of interest, and some of these genotyped plants will also be assessed with their trait performance as a training set (**Figure 4**). These marker and trait data in the training set can be analyzed using existing marker prediction models for GS such as RR-BLUP or Bayes-B implemented in various R packages (Endelman, 2011; Pérez-Rodriguez and de los Campos, 2014) to estimate marker “effects.” The estimated marker “effects” will be utilized to predict these breeding values in the prediction set for genetic ranking of parental lines.

The advantage of this alternative over regular GS mainly lies in the potential gains in marker prediction of individual trait performance through the application of FAST SNP markers. Realizing the gain in trait prediction highly depends on the development of the FAST SNP markers for individual traits, and requires further empirical investigations in breeding programs. To facilitate the development of FAST SNP markers through RNA-Seq (**Figure 3**), we proposed a new procedure, following the principle of BSR-Seq developed by Liu S. et al. (2012) and the methods used by Salem et al. (2012) and Ramirez-Gonzalez et al. (2015). However, it differs from using multiple pairs of individual plants with extreme trait values. We reasoned that the use of multiple pairs should be more powerful than bulking, as it can address not only many issues associated with BSR-Seq such as replication, but also, more importantly, increase the power of identifying consensus differentially expressed transcripts and the accuracy of putative SNP discovery with allelic differential (**Figure 3**). We suggest 10 or more pairs for the effort, but the optimum pairs to be used remain to be empirically determined and they may vary with respect to trait and plant mating system.

However, issues are not lacking in the development and application of FAST SNP markers through RNA-Seq. A complete set of genome wide function-associated SNP markers can be effectively generated through exome capture technology (Mascher et al., 2013; Warr et al., 2015) simultaneously for many traits, but not necessarily specified for a trait of interest. RNA-Seq can produce FAST SNP markers, but these markers may not be comprehensive for the trait, as gene expressions have spatio-temporal specificity. Successful identification of SNP markers associated with casual genes will depend on the gene expression in related tissues over different development stages, so tissue selection and sampling for RNA collection are critical and may vary in effectiveness for different traits. Our proposal (**Figure 3**) did not consider the multiple developmental stages of RNA sampling to capture all expressed genes associated with the trait performance and may miss some trans-regulatory genes (Druka et al., 2010). Some quantitative traits such as yield, maturity and disease resistance may need more research effort and proper experimental design to sample genes expressed at different developmental stages. Also, research efforts to develop FAST SNP markers for different traits may vary, as the genetic basis of different traits may differ. Uncertainty may also exist in the informativeness of RNA-Seq FAST SNP markers developed in one population for their applicability into other populations. Moreover, developing FAST SNP markers may be more complicated and more consideration may be needed in outcrossing, than selfing, plant species, as the genetic background for a trait in outcrossing plants is more heterogeneous.

In spite of these issues, FAST SNP markers for specific traits can be developed for plant breeding, either following our proposed procedure (**Figure 3**) or using existing methods such as eQTL analysis, GWAS, or those methods used in fish breeding (e.g., Salem et al., 2012). The good examples are the successful developments of FAST SNP markers through RNA-Seq in fish (e.g., see Salem et al., 2012; Ulloa et al., 2015) and 175 putative SNP markers associated with *Yr15*, a major disease resistance gene for wheat yellow rust (Ramirez-Gonzalez et al., 2015). Built upon these leading efforts, our derived alternative will provide an option for plant breeders with new procedures to develop and focus on a set of FAST SNP markers for trait prediction to enhance parental selection. Even with a small number of FAST SNP markers available for a given trait, our alternative is still applicable and may yield more informative parental selection than those with the aid of individual QTL markers in traditional MAS. Also, our synthesis is encouraging, as continuous search for better alternatives based on the other genetic characteristics of a quantitative trait is possible and may be more fruitful to provide much needed accuracy in marker-based prediction of a quantitative trait for molecular plant breeding.

## Concluding Comments

Our search for a better marker-based prediction of trait performance through literature review and theoretical reasoning yielded an alternative to regular genome selection for individual trait prediction. More accurate trait predictions can be theoretically achieved through the development of FAST SNP markers with RNA-Seq technique and the application of these markers to genotype plants and to predict breeding values following existing genomic prediction methods in breeding populations. Further empirical investigation is needed to realize how much gain in trait prediction with respect to breeding efficiency could be achieved from the derived alternative in a plant breeding program. The derived alternative may be questioned for its breeding efficiency in multiple-traits breeding, as function-associated SNP markers unspecified for specific traits could be more efficiently developed from exome capture technology than the proposed FAST SNP markers. However, our synthesis is encouraging, as continuous search for better alternatives based on the other genetic characteristics of a quantitative trait is possible and may yield more accurate trait prediction for molecular plant breeding.

## Ethics Statement

The writing process of this manuscript complies with the current laws of Canada.

## Author Contributions

Y-BF conceived of the research, conducted the literature review, performed the computer simulation and wrote the paper. M-HY conducted the literature review, performed the computer simulation and revised the paper. FZ conducted the literature review and wrote the paper. BB conducted the literature review and revised the paper.

## Conflict of Interest Statement

The authors declare that the research was conducted in the absence of any commercial or financial relationships that could be construed as a potential conflict of interest.
