# Supplementary material for: Searching for an Accurate Marker-Based Prediction of an Individual Quantitative Trait in Molecular Plant Breeding
Source: Front Plant Sci. 2017 Jul 6;8:1182. doi: 10.3389/fpls.2017.01182 (PMC5498511; doi:10.3389/fpls.2017.01182)
Supplement: Supplementary file 1 [file Tables_1_and_2.pdf]

Supplementary material for

Yong-Bi Fu, Mo-Hua Yang, Fangqin Zeng, and Bill Biligetu (2017) Searching for an accurate marker-based prediction of an individual quantitative trait in molecular plant breeding. Frontiers in Plant Science. DOI: 10.3389/fpls.2017.01182

1. **TABLE S1 |** A list of 187 prediction accuracies in 31 publications.
2. **TABLE S2 |** Simulation results on the correlations between true and predicted genotypic values in the validation data set for a quantitative trait with heritabilities 0.5 and 0.2 by three genomic selection models (RR-BLUP, Bayes-C or Bayes-B) based on 36,543 soybean SNP data with respect to QTL scenario and marker panel.

TABLE S1 | A list of 187 prediction accuracies in 31 publications

**Reference**	**Species**	**Predicted traits**	**Prediction accuracy**
Annicchiarico et al. (2015)	Alfalfa	biomass yield	0.35
		biomass yield	0.32
Arruda et al. (2016)	wheat (*Triticum aestivum* L.)	severity (SEV)	0.38
		incidence (INC)	0.52
		FHB index (FHBdx)	0.39
		Fusarium-damaged kernel (FDK)	0.77
		incidence-severity-kernel index (ISK)	0.64
		mycotoxin accumulation (DON)	0.61
Ashraf et al. (2016)	wheat (*Triticum aestivum* L.)	plant height	0.42
		maturity	0.4
		heading date	0.4
		grain yield	0.25
dos Santos et al. (2016)	maize	PRK	0.569
		ERIS	0.241
Duangjit et al. (2016)	tomato	ASA	0.601
		Asparagine	0.449
		Aspartate	0.126
		Beta-alanine	0.413
		Citrate	0.246
		DHA	0.658
		Erythritol	0.621
		Fructose	0.594
		Fucose	0.438
		GABA	0.639
		Galacturonate	0.439
		Glucuronate	0.363
		Glutamate	0.522
		Glutamine	0.478
		Glycerol-3P	0.444
		Inositol-1P	0.432
		Lysine	0.21
		Malate	0.519
		Maltitol	0.674
		Maltose	0.471
		Methionine	0.239
		Nicotinate	0.705
		2-Oxoglutarate-	0.509
		Phenylalanine	0.352
		Proline	0.709
		Putrescine	0.473
		Rhamnose	0.529
		Saccharate	0.309
		Serine	0.229

		Sucrose	0.571
		Threonate	0.052
		Threonine	0.363
		Tocopherol	0.451
		Tyramine	0.604
		Xylose	0.376
		a*	0.567
		b*	0.581
		Firmness	0.614
		FW	0.814
		L	0.617
		LN	0.423
		pH	0.506
		Soluble solid	0.714
		Sugar content	0.649
		TA	0.619
El-Dien et al. (2015)	interior spruce [white spruce (*Picea glauca* (Moench) Voss), Engelmann spruce (*Picea engelmannii* Parry), and their hybrids]	(height in m) HT	0.45
		diameter at breast height in cm (DBH)	0.4
		stem volume in m^3 (VOL)	0.41
		acoustic velocity in km/s (VDir)	0.49
		resistance to drilling (WDRes)	0.46
		wood density in kg/m^3 (WDX-ray)	0.5
		dynamic modulus of elasticity (MoEd)	0.48
Grenier et al. (2015)	Rice (*Oryza sativa* L.)	flowering time (FL)	0.192
		plant height (PH)	0.489
		grain yield (YLD)	0.239
		panicle weight (PW)	0.274
He et al. (2016)	winter wheat (*Triticum aestivum* L.)	Grain yield	0.65
Huang et al. (2016)	winter wheat (*Triticum aestivum* L.)	YLD(kg/ha-1)	0.33
		TW(kg/m-3)	0.66
		HGT(cm)	0.54
		HD(Julian day)	0.56
		FY(%)	0.49
		FP(%)	0.41
		SU(%)	0.6
		LA(%)	0.64
		WA(%)	0.65
		SO(%)	0.57
		SE(%)	0.37
Isidro et al. (2015)	Wheat (*Triticum aestivum* L.)	grain yield	0.463
		test weight	0.44
		lodging	0.35
		heading date	0.583
		plant height	0.51
	Rice (*Oryza sativa* L.)	florets per panicle	0.578
		flowering time	0.624
		plant height	0.69
		protein content	0.415
Isik et al. (2016)	maritime pine (*Pinus pinaster* Ait.)	stem sweep	0.49
		height	0.47
		tree diameter	0.43
Iwata et al. (2015)	Rice (*Oryza sativa* L.)	grain shape (A)	0.42
		grain shape (B)	0.59
		grain shape (C)	0.64
Jan et al. (2016)	Canola (*Brassica napus*)	seedling emergence	0.29
		days to flowering	0.56
		lodging resistance	0.39
		oil yield	0.75

		seed yield	0.45
		seed oil content	0.81
		seed glucosinolate content	0.61
Jiang and Reif (2015)	Wheat-1a	GY_E1	0.505
		GY_E2	0.493
		GY_E3	0.379
		GY_E4	0.484
	Wheat-2b	GY_drought	0.435
		GY_irrigated	0.537
	Maize-1c	GY_drought	0.429
		GY_irrigated	0.537
	Maize-2 d dent	DMY	0.632
	Maize-2 d flint	DMY	0.651
Lado et al. (2016)	wheat (*Triticum aestivum* L.)	Grain yield	0.5
Li et al. (2015a)	Rapeseed (*Brassica napus* L.)	flowering time	0.638
Li et al. (2015b)	Alfalfa (*Medicago sativa* L.)	Biomass yield	0.43
		Biomass yield	0.45
		Biomass yield	0.26
Liu et al. (2016)	Bread wheat (*Triticum aestivum* L.)	Gluten content	0.37
		Kernel hardness	0.34
		Protein content	0.36
		SDS value	0.48
		Starch content	0.27
		Test weight	0.41
		1000-kernel weight	0.39
Michel et al. (2016)	bread wheat (*Triticum aestivum* L.)	grain yield	0.38
		protein content	0.51
		protein yield	0.16
Mirdita et al. (2015)	winter wheat	FHB	0.6
		STB	0.5
Oakey et al. (2016)	barley (*Hordeum vulgare* L.)	height (H)	0.366
Onogi et al. (2015)	Asian rice (*Oryza sativa* L.)	days to heading (DH)	0.81
		culm length (CL)	0.79
		panicle length (PL)	0.61
		panicle number (PN)	0.42
		grain length (GL)	0.43
		grain width (GW)	0.68
		brown rice length (BL)	0.58
		brown rice width (BW)	0.57
Pace et al. (2015)	maize (*Zea mays* L.)	total root length (TRL)	0.54
		secondary root length (SEL)	0.32
		primary root length (PRL)	0.31
Pierre et al. (2016)	spring wheat (*Triticum aestivum* L.)	grain yield (GY)	0.36
		days to anthesis (DTA)	0.78
		days to maturity (DTM)	0.57
		plant height (PH)	0.76
Ramstein et al. (2016)	Switchgrass (*Panicum virgatum* L.)	dry matter yield (DMY)	0.499
		plant height (PH)	0.461
		heading date (HD)	0.41
Schmidt et al. (2016)	spring barley (*Hordeum vulgare* L.)	AMA	0.444
		AMB	0.142
		EXT	0.558
		FAN	0.497
		Fin_At	0.495
		FRI	0.552
		GLU	0.479
		KOL	0.487
		LOSS	0.419
		NIT	0.583
		PRT	0.521
		VIS	0.449
	winter barley(*Hordeum vulgare* L.)	AMA	0.564

		AMB	0.606
		EXT	0.625
		FAN	0.572
		Fin_At	0.732
		FRI	0.788
		GLU	0.798
		KOL	0.556
		LOSS	0.652
		NIT	0.551
		PRT	0.399
		VIS	0.744
Schulthess et al. (2016)	rye (*Secale cereale* L.)	grain yield (GY)	0.52
		protein content (PC)	0.32
Spindel et al. (2015)	Rice (*Oryza sativa* L.)	grain yield (GY)	0.31
		plant height (PH)	0.34
		flowering time (FL)	0.63
Tayeh et al. (2015)	Pea	Thousand seed weight(TSW)	0.83
		the number of seeds per plant (NSeed)	0.68
		the date of flowering (BegFlo)	0.65
Velu et al. (2016)	Wheat (*Triticum aestivum* L.)	grain Zn concentration (GZnC)	0.542
		grain Fe concentration (GFeC)	0.529
		thousand-kernel weight (TKW)	0.51
		days to maturity (DTM)	0.539
Zhang et al. (2016)	soybean (*Glycine max*)	seed weight (SW)	0.74

References

Annicchiarico P, Nazzicari N, Li X et al (2015) Accuracy of genomic selection for alfalfa biomass yield in different reference populations. BMC genomics 16:1020

Arruda MP, Lipka AE, Brown PJ et al (2016) Comparing genomic selection and marker-assisted selection for Fusarium head blight resistance in wheat (Triticum aestivum L.). Mol Breeding 36:84

Ashraf B, Edriss V, Akdemir D et al (2016) Genomic prediction using phenotypes from pedigreed lines with no marker data. Crop Sci 56:957-964.

dos Santos JP, Pires LP, de Castro Vasconcellos RC et al (2016) Genomic selection to resistance to Stenocarpella maydis in maize lines using DArTseq markers. BMC genetics 17: 86

Duangjit J, Causse M, Sauvage C (2016) Efficiency of genomic selection for tomato fruit quality. Mol Breeding 36:29

El-Dien OG, Ratcliffe B, Klápšte J et al (2015) Prediction accuracies for growth and wood attributes of interior spruce in space using genotyping-by-sequencing. BMC genomics 16:370

Grenier C, Cao TV, Ospina Y et al (2015) Accuracy of genomic selection in a rice synthetic population developed for recurrent selection breeding. PloS One 10:e0136594

He S, Schulthess AW, Mirdita V et al (2016) Genomic selection in a commercial winter wheat population. Theor Appl Genet 129: 641-651

Huang M, Cabrera A, Hoffstetter A et al (2016) Genomic selection for wheat traits and trait stability. Theor Appl Genet 129: 1697-1710

Isidro J, Jannink JL, Akdemir D et al (2015) Training set optimization under population structure in genomic selection. Theor Appl Genet 128:145-158

Isik F, Bartholomé J, Farjat A et al (2016) Genomic selection in maritime pine. Plant Sci 242:108-119

Iwata H, Ebana K, Uga Y et al (2015) Genomic prediction of biological shape: elliptic fourier analysis and kernel partial least squares (PLS) regression applied to grain shape prediction in rice (Oryza sativa L.). PloS One 10:e0120610

Jan HU, Abbadi A, Lücke S et al (2016) Genomic prediction of testcross performance in canola (Brassica napus). PloS One 11: e0147769

Jiang Y, Reif JC (2015) Modeling epistasis in genomic selection. Genetics 201:759-768

Lado B, Barrios PG, Quincke M et al (2016) Modeling genotype× environment interaction for genomic selection with unbalanced data from a wheat breeding program. Crop Sci 56:1-15

Li L, Long Y, Zhang L et al (2015) Genome wide analysis of flowering time trait in multiple environments via high-throughput genotyping technique in Brassica napus L. PloS One 10 :e0119425

Li X, Wei Y, Acharya A et al (2015) Genomic prediction of biomass yield in two selection cycles of a tetraploid alfalfa breeding population. Plant Genome 8(2)

Liu G, Zhao Y, Gowda M et al (2016) Predicting hybrid performances for quality traits through genomic-assisted approaches in Central European Wheat. PloS One 11:e0158635

Michel S, Ametz C, Gungor H et al (2016) Genomic selection across multiple breeding cycles in applied bread wheat breeding. Theor Appl Genet 129:1179-1189

Mirdita V, He S, Zhao Y et al (2015) Potential and limits of whole genome prediction of resistance to Fusarium head blight and Septoria tritici blotch in a vast Central European elite winter wheat population. Theor Appl Genet 128:2471-2481

Oakey H, Cullis B, Thompson R et al (2016) Genomic selection in multi-environment crop trials. G3: Genes Genomes Genetics 6:1313-1326

Onogi A, Ideta O, Inoshita Y et al (2015) Exploring the areas of applicability of whole-genome prediction methods for Asian rice (Oryza sativa L.). Theor Appl Genet 128:41-53

Pace J, Yu X, Lübberstedt T (2015) Genomic prediction of seedling root length in maize (Zea mays L.). Plant J 83:903-912

Pierre CS, Burgueño J, Crossa J et al (2016) Genomic prediction models for grain yield of spring bread wheat in diverse agro-ecological zones. Sci Rep 6:27312

Ramstein GP, Evans J, Kaeppler SM et al (2016) Accuracy of genomic prediction in Switchgrass (Panicum virgatum L.) improved by accounting for linkage disequilibrium. G3: Genes Genomes Genetics 4:1049-1062

Schmidt M, Kollers S, Maasberg-Prelle A et al (2016) Prediction of malting quality traits in barley based on genome-wide marker data to assess the potential of genomic selection. Theor Appl Genet 129:203-213

Schulthess AW, Wang Y, Miedaner T et al (2016) Multiple-trait-and selection indices-genomic predictions for grain yield and protein content in rye for feeding purposes. Theor Appl Genet 129:273-287

Spindel J, Begum H, Akdemir D et al (2015) Genomic selection and association mapping in rice (Oryza sativa). PloS Genet 11:e1004982

Tayeh N, Klein A, Le Paslier MC et al (2015) Genomic prediction in pea: effect of marker density and training population size and composition on prediction accuracy. Front Plant Sci 6:941

Velu G, Crossa J, Singh RP et al (2016) Genomic prediction for grain zinc and iron concentrations in spring wheat. Theor Appl Genet 129:1595-1605

Zhang J, Song Q, Cregan PB et al (2016) Genome-wide association study, genomic prediction and marker-assisted selection for seed weight in soybean (Glycine max). Theor Appl Genet 129:117-130

TABLE S2 | Simulation results on the correlations between true and predicted genotypic values in the validation data set for a quantitative trait with heritability of 0.5 and 0.2 by three genomic prediction models (RR-BLUP, Bayes-C or Bayes-B) based on 36,543 soybean SNP data with respect to QTL scenario and marker panel.

	Heritability=0.5[#]		Heritability=0.2[#]		
QTL scenario/marker panel[ϕ]	RR-BLUP	Bayes-C	RR-BLUP	Bayes-C	Bayes-B
QTL50					
mp1: 50 QTL	0.94(0.01)	0.94(0.01)	0.83(0.04)	0.81(0.04)	0.82(0.04)
mp2: 50 QTL + 50 HLD	0.92(0.01)	0.93(0.01)	0.81(0.04)	0.81(0.04)	0.81(0.04)
mp3: 36543 SNPs with QTL	0.62(0.08)	0.62(0.08)	0.53(0.13)	0.52(0.13)	0.53(0.13)
mp4: 50 HLD	0.84(0.03)	0.83(0.03)	0.73(0.06)	0.72(0.06)	0.72(0.06)
mp5: 36543 SNPs - 50 QTL	0.61(0.08)	0.61(0.08)	0.52(0.13)	0.52(0.13)	0.52(0.13)
mp6: 100 HLDr2	0.73(0.05)	0.73(0.06)	0.62(0.09)	0.62(0.09)	0.63(0.09)
mp7: 100 HLDr2 + 50 rSNP	0.71(0.06)	0.72(0.06)	0.59(0.10)	0.60(0.10)	0.61(0.10)
QTL100					
mp1: 100 QTL	0.89(0.02)	0.88(0.02)	0.73(0.06)	0.71(0.07)	0.72(0.07)
mp2: 100 QTL + 100 HLD	0.87(0.02)	0.87(0.02)	0.71(0.06)	0.70(0.06)	0.70(0.07)
mp3: 36543 SNPs with QTL	0.60(0.08)	0.60(0.08)	0.50(0.10)	0.50(0.11)	0.50(0.10)
mp4: 100 HLD	0.78(0.04)	0.77(0.04)	0.65(0.06)	0.63(0.08)	0.63(0.07)
mp5: 36543 SNPs - 100 QTL	0.60(0.08)	0.60(0.08)	0.50(0.11)	0.50(0.11)	0.50(0.11)
mp6: 200 HLDr2	0.67(0.08)	0.67(0.07)	0.56(0.08)	0.56(0.09)	0.56(0.09)
mp7: 200 HLDr2 + 100 rSNP	0.65(0.08)	0.66(0.08)	0.54(0.09)	0.54(0.10)	0.55(0.10)
QTL250					
mp1: 250 QTL	0.79(0.03)	0.79(0.03)	0.61(0.09)	0.60(0.09)	0.60(0.09)
mp2: 250 QTL + 250 HLD	0.77(0.04)	0.77(0.04)	0.60(0.09)	0.60(0.09)	0.59(0.09)
mp3: 36543 SNPs with QTL	0.61(0.07)	0.61(0.07)	0.49(0.11)	0.49(0.11)	0.49(0.11)
mp4: 250 HLD	0.71(0.05)	0.71(0.05)	0.56(0.09)	0.55(0.09)	0.56(0.09)
mp5: 36543 SNPs - 250 QTL	0.61(0.07)	0.61(0.07)	0.49(0.11)	0.49(0.11)	0.48(0.11)
mp6: 500 HLDr2	0.63(0.06)	0.64(0.06)	0.51(0.11)	0.51(0.11)	0.51(0.11)
mp7: 500 HLDr2 + 250 rSNP	0.63(0.06)	0.62(0.06)	0.51(0.11)	0.51(0.11)	0.51(0.11)
QTL500					
mp1: 500 QTL	0.71(0.06)	0.71(0.06)	0.56(0.07)	0.55(0.07)	0.55(0.07)
mp2: 500 QTL + 500 HLD	0.70(0.06)	0.70(0.06)	0.55(0.07)	0.55(0.07)	0.55(0.07)
mp3: 36543 SNPs with QTL	0.60(0.08)	0.60(0.08)	0.48(0.07)	0.48(0.07)	0.48(0.07)
mp4: 500 HLD	0.66(0.07)	0.66(0.08)	0.53(0.07)	0.53(0.07)	0.53(0.07)
mp5: 36543 SNPs - 500 QTL	0.60(0.08)	0.60(0.08)	0.48(0.07)	0.48(0.07)	0.48(0.07)
mp6: 1000 HLDr2	0.62(0.08)	0.62(0.08)	0.50(0.07)	0.49(0.07)	0.50(0.08)
mp7: 1000 HLDr2 + 500 rSNP	0.61(0.08)	0.62(0.08)	0.49(0.07)	0.49(0.07)	0.49(0.07)

[ϕ] For each of four QTL scenarios, mp1 is an ideal case with only QTL genotypes; mp2 is another extreme with both QTL genotypes and equal number of marker loci with the highest linkage disequilibrium (HLD) for each QTL; mp3 reflects the common practice with all genome-wide SNPs, including QTL; mp4 represents a case of markers each having HLD with an QTL; and mp5 reflects a case of markers with all the SNPs minus QTL; mp6 consists of the marker loci in which two markers are randomly selected from loci with the highest 20 LD values for each QTL (HLDr2), and mp7 considers both mp6 and a set of random SNP markers (rSNP) each falsely representing an QTL.

[#] The training and validation populations consisted of 400 randomly selected plants each. The results by Bayes-B for heritability 0.5 are shown in Table 1. The standard deviations of the correlations are given in parentheses.
